# The effect of the health transformation plan on cesarean section in Iran: a systematic review of the literature

**DOI:** 10.1186/s13104-019-4081-y

**Published:** 2019-01-18

**Authors:** Meysam Behzadifar, Masoud Behzadifar, Ahad Bakhtiari, Samad Azari, Mandana Saki, Farnaz Golbabayi, Nicola Luigi Bragazzi

**Affiliations:** 1 Social Determinants of Health Research Center, Lorestan University of Medical Sciences, Khorramabad, Iran; 2 Health Management and Economics Research Center, Iran University of Medical Sciences, Tehran, Iran; 3 Department of Health Management and Economics, School of Public Health, Tehran University of Medical Science, Tehran, Iran; 4 Nursing Care Research Center (NCRC), School of Nursing and Midwifery, Iran University of Medical Sciences, Tehran, Iran; 5 School of Public Health, Department of Health Sciences (DISSAL), University of Genoa, Genoa, Italy

**Keywords:** Cesarean section, Health transformation plan, Health policy, Iran

## Abstract

**Objective:**

Cesarean section (CS) is one of the most common surgical procedures in the world. In developed and developing countries, CS has grown significantly over the past decades. The Iranian Ministry of Health and Medical Education has developed a health transformation plan (HTP) in order to reduce CS rate and promote vaginal delivery. This study was conducted with the aim of reviewing the results of published studies on the impact of the HTP on CS in Iran.

**Results:**

We searched Embase, PubMed/MEDLINE, ISI/Web of Sciences, Scopus, as well as Iranian databases (MagIran, SID and Barakatkns), from May 2014 to October 2018. To assess the quality of studies, the checklist “A Cochrane Risk of Bias Assessment Tool: for Non-Randomized Studies of Interventions” was utilized. Twelve studies were selected. Seven studies reported statistically significant results, showing a positive impact of the implementation of the HTP on CS reduction. Despite the decreased CS rate in Iran after about 4 years of the implementation of this policy, the goal of a yearly reduction by 10% has not been achieved yet. Increasing access to maternity services and community-based education through mass media could help changing the attitudes of Iranian mothers towards CS.

## Introduction

Health is a valuable global capital, and, hence, health policy- and decision-makers are working to improve it, ensuring the delivery of better health provisions, a fair and just access to healthcare facilities and implementing effective policies [1]. Most countries are developing, evaluating and re-programming health policies, and, therefore, health system reforms can be considered as a continuous improvement cycle to achieve better health levels [2]. In its 2000 report, the World Health Organization (WHO) has stated that public organisms and organizations should promote health and well-being, meeting with the expectations of the users, guaranteeing access to services and protecting against increasing health costs [3].

Iran, one of the Eastern Mediterranean Regional Office (EMRO) countries, has suffered from various health problems due to economic-financial issues, causing people to be dissatisfied with the services of various sectors of the healthcare system. Following the establishment of President Rouhani, the Ministry of Health and Medical Education (MoHME) of Iran has developed a health sector evolution plan (“health transformation plan”, HTP). The HTP, which has sought to financially protect people against healthcare costs, has significantly improved the quality of health services and has increased their access. It has been implemented in public hospitals nationwide since May 5, 2014 [4].

One of the indicators of proper healthcare provisions is the rate of cesarean section (CS). CS is one of the most common surgical procedures worldwide. In developed and developing countries, CS has grown significantly over the past decades [5, 6]. In 1985, the WHO announced that the acceptable rate of CS should be in the range 5–15% [7]. According to a meta-analysis, CS rate in Iran was estimated to be 48% [8]. This high, unacceptable figure can be seen as a warning, which should foster the implementation of adequate policies to reduce CS in Iran.

Unnecessary CS can have adverse consequences on health both for the mother and the infant. A WHO study showed that maternal mortality was higher for CS than for vaginal delivery [9], also indicating that there is a very strong relationship between CS and infant mortality [10]. Furthermore, unnecessary CS imposes financial costs both for households and the entire healthcare system [5].

Health policy- and decision-makers in Iran in the past have proposed several plans to reduce CS rate [11]. Lack of proper knowledge of the complications of CS, fear of pain, psychological stress, and shortened delivery time are the major factors contributing to the choice of having CS [12, 13].

A comprehensive package for the promotion of vaginal delivery aimed at promoting maternal and infant health in public hospitals was included in the HTP. According to this policy, all hospitals were required to curb the CS rate by 10% per year. In order to encourage mothers to undergo normal vaginal delivery, this was offered in public state hospitals free of charge. In addition, to protect the mother’s privacy and the pleasantness of the delivery process, the environment, in which the delivery was performed, was optimized. Encouraging public agencies and service providers to deliver methods for reducing labor pain, including pharmacological and non-pharmacological methods, was also implemented by the plan. In addition, to support the culture of pregnancy and childbirth, the provision of maternity-ready classes for pregnant mothers and the empowerment of service providers were among the other measures taken for the promotion of vaginal delivery. To investigate the effect of the HTP on CS after its implementation, different studies have been carried out. Pooling these investigations together can help assessing the overall effect of the HTP on CS rate, as well as improving decision making for developing and providing more suitable and effective programs. Therefore, this study was conducted with the aim of systematically reviewing the results of published studies on the impact of the HTP on CS in Iran.

## Main text

### Literature search

This review has been performed according to the “The Meta-analysis of Observational Studies in Epidemiology” (MOOSE) guidelines [14]. Two authors independently searched several scholarly databases, including Embase, PubMed/MEDLINE, ISI/Web of Sciences (WOS), Scopus, as well as Iranian thesauri (such as MagIran, SID and Barakatkns), from May 2014 to October 2018. The search strategy was: (“cesarean” OR “cesarean section” OR “caesarean delivery” OR “childbirth”) AND (“health system reform” OR “health reform” OR “health sector evolution plan” OR “health transition” OR “health transition plan” OR “health transformation” OR “health transformation plan”) AND (Iran). The search strategy was developed by consulting an expert librarian. The reference list of each eligible article was also reviewed for potentially relevant studies. Any disagreements were resolved through discussion.

### Inclusion and exclusion criteria

Studies investigating the effect of the HTP on CS in the form of cross-sectional, cohort, time-series studies written in English or Persian were included. Those studies whose results were not clear, or designed as letters to editor, editorials, case-reports, case-series, commentaries or conference abstracts were excluded.

### Quality assessment

To assess the quality of studies included in the present systematic review, the checklist “A Cochrane Risk of Bias Assessment Tool: for Non-Randomized Studies of Interventions” (ACROBAT-NRSI) was utilized [15].

### Data extraction

The surname of the first author, the year of publication, the location of the study, the study design, the number of participants, and the most important findings of the included studies were independently extracted by 2 authors. Any discrepancy was solved through discussion.

## Results

**Fig. 1:**
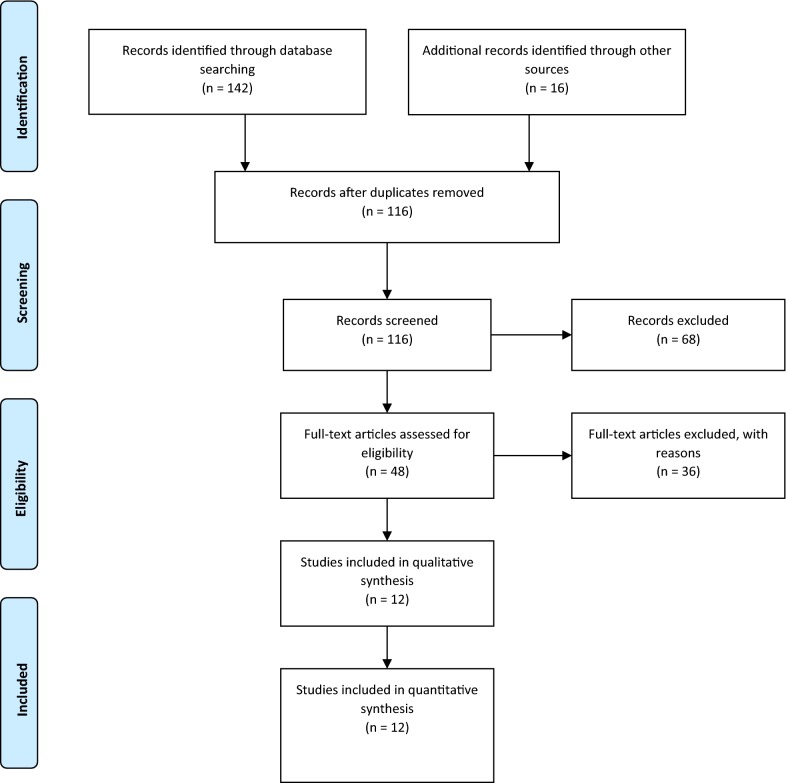
Process of studies retrieval and selection

In the initial search, 158 studies were found. Then, 42 duplicate studies were deleted. After reviewing the title, 68 studies were excluded. In the next step, the abstract of the studies was assessed and, finally, 12 studies meeting with the previously stated inclusion criteria were selected [16–27], as shown in Fig. 1.

Studies were conducted between 2015 and 2018. In most studies, the HTP was implemented in public hospitals, whereas in 3 and 2 studies in private and health ministry hospitals, respectively. According to the study design, 4 and 6 investigations were cross-sectional and descriptive-analytic studies, 1 was a time series analysis and 1 a quasi-experimental investigation. Seven studies reported statistically significant results. An overall decrease in CS rate was observed for public hospitals, whereas the effect of the HTP on CS in private hospitals was mixed, with 1 study reporting a significant increase, 1 study computing a significant decrease and a further study reporting unclear findings (Table 1).

**Table 1:** The main findings of the studies included in the present systematic review

First author/references	Year of publication	Location	Study design	Method of study	Main findings
Zarei [16]	2015	Tehran	Cross-sectional	A public hospital was selected and information from 2013 and 2014 was compared	Physicians were able to reduce CS by 3 to 7% compared to last year
Piroozi [17]	2016	Kurdistan	Descriptive-analytic	Data were collected in 2013 and 1 year after the implementation of the HTP in public hospitals, Social Security Hospitals and 1 private hospital	CS decreased by 14.62% compared to the year before the plan was implemented in 9 public state hospitals. In social welfare hospitals, CS rate worsened in 2 hospitals. In the private hospital, a 0.21% increase in CS was observed. Results were statistically significant
Seidali [18]	2016	Khuzestan	Descriptive-analytic	Data were collected 6 months after the implementation of the plan and compared with the previous year in public hospitals	After implementation of the HTP, CS rate decreased from 49.56% to 32.10%. Results were statistically significant
Rooeintan [19]	2016	Fars	Cross-sectional	Information was collected from private and public hospitals between 2013 and 2014	CS dropped from 64.7% to 58.6%. Findings about the decrease or increase in private hospitals were unclear. The results were not statistically significant
Dehghan [20]	2017	Yazd	Descriptive-analytic	Information was collected and compared between 2013 and 2014 in 15 public and private hospitals	CS decreased from 52.64% to 47.37%. In public hospitals, it decreased from 45.2% to 36.71% and in private hospitals from 56.7% to 54.36%. Results were statistically significant
Fouladi [21]	2017	Qom	Descriptive-analytic	Two public hospitals were selected and data were compared 2 years before and 2 years after the implementation of the plan	The rate of CS in hospital A decreased from 49.43% to 41% and in hospital B from 46.76% to 43.36%. Results were not statistically significant
Zaboli [22]	2017	Kerman	Cross-sectional	Seven public hospitals were selected and information was reviewed 6 months before the plan and 6 months after	CS decreased from 48.02% to 43.43%. Results were not statistically significant
Zandian [23]	2017	Ardabil	Cross-sectional	Information between 2013 and 2015 was collected from a public hospital	CS decreased from 60.5% to 43%. Results were statistically significant
Karami Matin [24]	2018	Kermanshah	Time series analysis	Information from 15 public hospitals was reviewed between 2012 and 2016	CS dropped by 11% a month after the implementation of the plan, and after the implementation of the plan, the monthly increase was 0.0017%. Results showed that the plan was not effective in reducing CS.
Rezaie [25]	2018	Fars	Descriptive-analytic	Between 2014 and 2015, information was collected from a public hospital	CS was reduced from 47.57% to 38.70%. Results were statistically significant
Yusefi [26]	2018	Fars	Descriptive-analytic	Information was collected from 10 public hospitals between 2013 and 2015	CS decreased. Results were statistically significant
Jabbari [27]	2018	Isfahan	Quasi-experimental	Data was collected from 22 public and 6 private hospitals in 6 months before and after the implementation of the HTP	CS decreased. Results were statistically significant

The quality of the studies based on the ACROBAT-NRSI is presented in Table 2. More in detail, bias due to confounding was low for all studies, whilst bias in selection of participants was generally low but moderate for 2 studies. Bias in measurement of interventions was moderate for 3 studies, as well as bias due to departures from intended intervention. Bias due to missing data was moderate for 4 studies. Bias in measurement of outcomes was moderate for 5 studies, whereas bias in selection of reported results resulted moderate for 6 studies.

**Table 2:** Result of the quality assessment of the studies included in the present systematic review

Study	Domains of bias						
	Bias due to confounding	Bias in selection of participants	Bias in measurement of interventions	Bias due to departures from intended interventions	Bias due to missing data	Bias in measurement of outcomes	Bias in selection of reported results
Zarei	Low risk	Moderate risk	Low risk	Low risk	Moderate risk	Low risk	Moderate risk
Piroozi	Low risk	Low risk	Low risk	Moderate risk	Low risk	Low risk	Low risk
Seidali	Low risk	Low risk	Low risk	Low risk	Moderate risk	Moderate risk	Low risk
Rooeintan	Low risk	Low risk	Moderate risk	Low risk	Moderate risk	Low risk	Moderate risk
Dehghan	Low risk	Low risk	Low risk	Low risk	Low risk	Moderate risk	Moderate risk
Fouladi	Low risk	Moderate risk	Moderate risk	Low risk	Low risk	Moderate risk	Moderate risk
Zaboli	Low risk	Low risk	Low risk	Low risk	Moderate risk	Low risk	Low risk
Zandian	Low risk	Low risk	Moderate risk	Low risk	Low risk	Moderate risk	Moderate risk
Karami Matin	Low risk	Low risk	Low risk	Low risk	Low risk	Low risk	Low risk
Rezaie	Low risk	Low risk	Low risk	Moderate risk	Low risk	Low risk	Low risk
Yusefi	Low risk	Low risk	Low risk	Moderate risk	Low risk	Moderate risk	Moderate risk
Jabbari	Low risk	Low risk	Low risk	Low risk	Low risk	Low risk	Low risk

## Discussion

To the best of our knowledge, this study is the first systematic review of the impact of the implementation of the HTP on the CS rate in Iran. According to a meta-analytical study in 2014, the rate of CS was estimated to be 48% [8], comparable with the rate computed for Lebanon (49%) [6], but higher than the rate found for Pakistan (9.2%) [28]. In a study published in 2016, the temporal trend of CS between 1990 and 2014 has been studied. The highest percentages were found in Latin America and the Caribbean, whereas in Asia, Oceania, Europe and Africa a rate of 15.1%, 14.1%, 13.8%, and 4.5% was computed, respectively. CS had a worrying upward trend in most countries of the world. Therefore, policy- and decision-makers should take effective health policies and strategies to reduce this trend [29].

Health policy-makers in Iran should be aware that CS is a surgical operation that has its own complications and may have long-term effects and serious consequences for future pregnancies in women [30, 31]. Promotion of vaginal delivery and reduction of CS are the main priorities of the MoHME [11], which aims at ensuring a high quality maternal care. Therefore, the HTP was designed to reduce the rate of CS.

The present review showed that CS exhibited a decreasing trend after the implementation of the HTP, also because vaginal delivery was offered free of charge. Paying attention to financial issues in health system reform is, indeed, very important, and, on the other hand, the cost of CS directly impacts on the cost of health care [32]. Increasing the offer of CS would increase direct payments and out-of-pocket (OOP) expenditure, dramatically weakening the sustainability of the healthcare system. In a study by Moradi and collaborators, assessing the effectiveness of the package for the promotion of vaginal delivery, midwives and physicians said that free-of-charge offer represented a major incentive for choosing natural delivery, besides the reduced maternity services tariffs [33]. On the other hand, doctors and providers of maternity services have less legal responsibilities in performing normal labor compared to CS [34, 35].

Despite the decreased CS rate, the target of a yearly reduction by 10% was not achieved, probably for a variety of reasons, not currently well understood, including cultural, social and economic factors [29]. In a study, for instance, mothers have expressed concerns and pain of normal labor, including fear of rupture, deformation and relaxation of the *genitalia* [36].

Furthermore, the findings of the present study showed that, after the implementation of the HTP, the rate of CS increased in some private hospitals. This is consistent with the literature and studies performed in other countries. For example, in a study conducted in Peru, after reforming the health sector, and changing the payment mechanisms, CS rate increased from 28 to 53% [37]. Also, another study in Uruguay showed that, due to increased payments to doctors in the private sector, CS rate was twice as high as in the public sector [38], due to financial incentives for physicians and reimbursement of costs by insurers [39–41]. In many cases, insurance covers the cost of CS in the private sector, which makes mothers not worried about the costs. In a meta-analysis, the results showed that mothers with private insurance had a greater tendency for choosing CS in the private sector [42].

## Conclusions

Overall, a positive impact of the implementation of the HTP on CS reduction was shown in the existing scholarly literature. However, despite the decreased CS rate in Iran, the goal of a yearly reduction by 10% has not been achieved yet.

### Recommendation

Extensive efforts should be made to properly implement health policies, and, in this regard, support should be granted to all stakeholders and groups that can contribute to the effective implementation. If the process of implementation of a policy is accompanied by a slowdown and encounters problems, negotiation, training and various strategies should be taken as proper measures and interventions [43].

Health policy- and decision-makers have implemented a package for promoting natural delivery and reducing CS rate. To further explore the effect of this policy, more studies are needed in all Iranian provinces in public and, especially, private hospitals. To achieve the goal of reducing CS, all individuals and groups should be involved. Encouraging physicians to perform vaginal delivery through reforming the payment mechanisms, and increasing access to maternity services and community-based education through mass media could help changing the attitude of Iranian mothers towards CS.

## Limitations

This study has some limitations that should be properly mentioned: there is a dearth of studies aimed at evaluating the effectiveness of this policy on the reduction of CS in many Iranian provinces, especially the provinces with the highest rates of CS. Also, there is a need of qualitative studies on the tendency of mothers to choose CS rather than normal delivery, as well as investigations related to the opinions of physicians and midwives after the implementation of the HTP. Most studies merely collected information from public hospitals and less from private hospitals. Another shortcoming of the present review is the publication bias, due to the fact that gray literature was not searched.

## Abbreviations

HTP: health transformation plan
CS: cesarean section
MoHME: Ministry of Health and Medical Education
ACROBAT-NRSI: A Cochrane Risk of Bias Assessment Tool: for Non-Randomized Studies of Interventions
WHO: World Health Organization
EMRO: Eastern Mediterranean Regional Office
OOP: out-of-pocket
